# Occupational Exposure to Blood Borne Viruses Among Healthcare Workers in a Tertiary Care Referral Hospital in Tehran

**DOI:** 10.5812/hepatmon.12201

**Published:** 2013-07-01

**Authors:** Shahnaz Sali, Muayad A. Merza, Davood Yadegarinia

**Affiliations:** 1 Infectious Diseases and Tropical Medicine Research Center, Shahid Beheshti University of Medical Sciences, Tehran, IR Iran; 2 Azadi Teaching Hospital, School of Medicine, Faculty of Medical Sciences, University of Duhok, Duhok, Iraq

**Keywords:** Needle Sticks, Blood Borne Pathogen, Healthcare Workers

*Dear Editor,*

Occupational exposure to blood-borne viruses (BBVs) is a major concern in the health care setting. The BBVs of primary concern are the human immunodeficiency virus (HIV), hepatitis B virus (HBV), and hepatitis C virus (HCV). Health care workers (HCWs) can be exposed to these viruses through needle-stick and other injuries through contaminated material with blood and other infectious body fluids. Nurses, health technicians, cleaning personnel and physicians are potentially targets for such accidents. The Center for Disease Control and Prevention (CDC) estimates that hospital-based healthcare personnel sustain an estimated 385,000 percutaneous injuries (needle-sticks and other sharp-devices) (1) annually. In Iran, the annual incidence of occupational exposure to BBVs is unknown due to the limited data in this area. The World Health Organization (WHO) estimates that more than two million injuries causes about 66,000 HBV, 16,000 HCV and about 1,000 HIV infections among 35 million HCWs each year (2). The key in reducing accidental exposure to BBVs is through promoting educational awareness of health care staff on suitability of HBV vaccination, in house training courses concerning preventive measures of such accidents and implementing proper postexposure prophylaxis (PEP) and following-up. Here, we reviewed case notes of 193 HCWs accidently exposed to BBVs over four-years, from January 2009 to January 2013. The data were reviewed in Labbafinejad hospital, which is a specialized 231 bed public tertiary care referral hospital with a total number of 915 HCWs who are potentially at accidental risk of acquiring BBVs. The study was approved by the ethics committee in the hospital. The reviewed HCWs cases (193) were classified into 75 nurses, 39 medical doctors, 35 cleaning personnel, 30 medical technicians (17 surgical operation technicians, 10 lab technicians and 3 radiology technicians), 13 nursing students and 1 medical student. In this study, the nurses were in top list of accidental exposure, which is in line with other studies (3, 4). Injuries were categorized as follows; 150 had needle-stick injuries, 22 cases had sharp cuts like scalpel and broken glasses and 21 cases had blood splashes on their eyes. Among these 193 cases, 132 HCWs were exposed to known serologically tested HIV, HBV and HCV negative patients, 24 had exposure to HBsAg positive patient, 5 had exposure to anti-HCV positive patients and two were exposed to HIV seropositive patient (one was co-infected with HCV infection). A total of 30 HCWs had occupational exposures to unknown sero-status patients. Anti-HBs antibody titer of the 24 HCWs, who had HBV exposure, was higher than 200 mIU/mL. Therefore, no intervention was done for them expect one who, despite being vaccinated had low anti-HBs titer. Thus, he was treated with hepatitis B immunoglobulin (HBIG) with another full course of hepatitis B vaccine, as routine schedule. Six patients were exposed to HCV seropositive patients; they underwent lab investigations, the result of that was normal for everyone. In these patients, follow up 2 weeks RT-PCR was negative. Thereafter, 1, 3, 6 months lab investigations were repeated and results were negative. One HCW was exposed to seropositive HIV patient, as the result of which he received PEP for HIV infection and underwent follow up and the result was negative. Another HCW was exposed to seropositive HIV coinfected with HCV. He also received PEP for HIV and underwent follow up lab investigation for HCV infection and lab results were negative. Hence, no further action was advised. PEP therapy has been shown to reduce the risk of transmission of HIV following accidental exposure by 80% (5). In conclusion, avoiding accidental occupational exposure to BBVs is an important safety issue, which can be achieved to some extent by health awareness of the problem and further by strengthening health control measures and providing a safe hospital work environment.

## References

[1] Panlilio AL, Orelien JG, Srivastava PU, Jagger J, Cohn RD, Cardo DM (2004). Estimate of the annual number of percutaneous injuries among hospital-based healthcare workers in the United States, 1997-1998.. Infect Control Hosp Epidemiol..

[2] Prüss-Ustün A, Rapiti E, Hutin Y (2003). Sharps injuries: global burden of disease from sharps injuries to health-care workers..

[3] Nagao Y, Baba H, Torii K, Nagao M, Hatakeyama K, Iinuma Y (2007). A long-term study of sharps injuries among health care workers in Japan.. Am J Infect Control..

[4] Tabak N, Shiaabana AM, Shasha S (2006). The health beliefs of hospital staff and the reporting of needlestick injury.. J Clin Nurs..

[5] (2001). Updated U.S. Public Health Service Guidelines for the Management of Occupational Exposures to HBV, HCV, and HIV and Recommendations for Postexposure Prophylaxis.. MMWR Recomm Rep..

